# The relationships between sleep and cognitive decline: a 4‐year cohort study based on a population in a rural area of Xi'an, China

**DOI:** 10.1002/alz70860_098316

**Published:** 2025-12-23

**Authors:** Beiyu Zhao, Suhang Shang, Liangjun Dang, Shan Wei, Ling Gao, Chen Chen, Meng Wei, Qiumin Qu, Jin Wang

**Affiliations:** ^1^ The First Affiliated Hospital of Xi’an Jiaotong University, Xi’an, China; ^2^ The First Affiliated Hospital of Xi'an Jiaotong University, Xi’an, China; ^3^ The First Affiliated Hospital of Xi'an Jiaotong University, Xi'an, Shaanxi, China; ^4^ The First Affiliated Hospital of Xi’an Jiaotong University, Xi'an, Shaanxi, China; ^5^ XI'AN JIAOTONG UNIVERSITY, XI'AN, China

## Abstract

**Background:**

Sleep facilitates the removal of brain metabolites and the recovery of brain function; however, the effect of sleep on cognitive decline has not been fully established. This study aimed to analyze the role of sleep on cognitive decline after 4 years in middle‐aged and elderly people in rural areas of Xi’an.

**Method:**

This is a population based cohort study. The data were collected from the “Cognitive impairment cohort of middle‐aged and elderly people in rural areas of Xi’an”. The cognitive normal population surveyed in 2016 were followed‐up in 2020. Sleep quality was evaluated using the Pittsburgh Sleep Quality Index (PSQI), including sleep quality, time to fall asleep, sleep duration, sleep efficiency, sleep disorders, hypnotic drugs and daytime dysfunction. Cognitive function was assessed using the Mini‐Mental State Examination (MMSE). Cognitive decline was defined as a drop of ≥2 points on the MMSE after 4‐years follow‐up. The relationship between sleep and cognitive decline was analyzed using univariate and multivariate analyses.

**Result:**

Among the 1290 subjects, the mean age was 57.07±9.15 years and 40.2% were men. 737(57.13%) had poor sleep. Univariate analysis revealed that the sleep efficiency was lower in the cognitive decline group compared to the cognitively stable group (1.26±1.22 vs. 1.41±1.22, *p* = 0.036). No significant association was found between cognitive decline and the other six PSQI subitem. The multifactorial logistic analysis suggested that cognitive decline was associated with the less efficient sleep compared to the more efficient sleep group (OR=1.316, 95%CI: 1.016‐1.705, *p* = 0.037).

**Conclusion:**

Low sleep efficiency was associated with higher risk of cognitive decline after 4 years. It suggested that less efficient sleep maybe a risk factor for cognitive impairment.

